# Software-guided (PREVEDEL) cognitive stimulation to prevent delirium in hospitalised older adults: study protocol

**DOI:** 10.1186/s12877-023-04189-2

**Published:** 2023-08-05

**Authors:** Maricel Garrido, Evelyn Álvarez, Felipe Salech, Verónica Rojas, Nicole Jara, José Ignacio Farías, Daniela Ponce de la Vega, Eduardo Tobar

**Affiliations:** 1 Departamento de Medicina, Servicio de Medicina Física y Rehabilitación, Hospital Clínico de La Universidad de Chile, Dr. Carlos Lorca Tobar #999, 8380456 Independencia-Santiago, Chile; 2 Departamento de Terapia Ocupacional y Ciencia de La Ocupación, Facultad de Medicina, Universidad de Chile, Av. Independencia #1027, 8380453 Independencia-Santiago, Chile; 3 Centro de Estudios en Neurociencia Humana y Neuropsicología, Facultad de Psicología, Universidad Diego Portales, Vergara #275, 8320000 Santiago-Santiago, Chile; 4 Centro de Investigación Clínica Avanzada (CICA), Hospital Clínico de La Universidad de Chile, Dr. Carlos Lorca Tobar #999, 8380456 Independencia-Santiago, Chile; 5 Sección de Geriatría, Hospital Clínico de La Universidad de Chile, Dr. Carlos Lorca Tobar #999, 8380456 Independencia-Santiago, Chile; 6 Centro de Gerociencia, Salud Mental y Metabolismo, GERO, Las Palmeras #3425, 7800003 Ñuñoa, Santiago, Chile; 7 Departamento de Medicina, Unidad de Pacientes Críticos, Hospital Clínico de La Universidad de Chile, Carlos Lorca Tobar #999, 8380456 Independencia-Santiago, Chile; 8 International Research Project for the Humanization of Intensive Care Units (HU-CI Project), Calle Cerceda #11, Collado -Villalba, 28400 Madrid, Spain

**Keywords:** Delirium, Non-pharmacological prevention, Older adults, Software, Cognitive stimulation

## Abstract

**Background:**

Delirium is a clinical condition characterised by acute and fluctuating deterioration of the cognitive state, generally secondary to an acute pathology. Delirium is associated with negative outcomes in older adults, such as longer hospitalisations, higher mortality, and short and medium-term institutionalisation. Randomised clinical trials have shown that delirium is preventable through non-pharmacological prevention measures, decreasing its incidence by 30–50%. These interventions include promoting physical activity, facilitating the use of glasses and hearing aids, cognitive stimulation, and providing frequent reorientation of time and space, among others. These measures are currently seldom applied in hospitals in Chile and around the world for reasons including the heavy workload of clinical staff, the lack of trained personnel, and in general the absence of a systematic implementation processes.

We developed a software called PREVEDEL, which includes non-pharmacological strategies such as cognitive stimulation, early mobilisation, orientation, and pain assessment. We propose a randomised clinical trial to evaluate whether cognitive stimulation guided by PREVEDEL software prevents delirium status (full/subsyndromal delirium) in hospitalised older adults.

**Method:**

A randomised controlled trial, with parallel, multicentre groups. We will recruite patients 65 years or older who have been hospitalised for less than 48 h in the general ward or the intermediate care units of four hospitals in Santiago, Chile. The participants in the intervention group will use a tablet with cognitive stimulation software for delirium prevention for five continuous days versus the control group who will use the tablet without the software.

We will evaluate the incidence, duration, density of delirium, subsyndromal delirium with the Confusion Assessment Method, cognitive with the Montreal Cognitive Assessment, and functional status with the Functional Independence Measure at discharge. Moreover, we will evaluate the adherence to prevention measures, as well as demographic variables of interest.

**Discussion:**

The use of cognitive PREVEDEL software could increase and improve the implementation of non-pharmacological prevention measures for delirium in hospitalised older adults, thus reducing its incidence and contributing to patients and health professionals.

**Trial registration:**

NCT05108207 ClinicalTrials.gov. Registered 4 November 2021.

## Background

Delirium is a clinical condition characterised by acute cognitive impairment, of fluctuating course, generally secondary to an acute pathology, which appears after the interaction between predisposing factors and well-described precipitants. It is a very common condition in older adults, observed in 20–30% of patients hospitalised in a general ward, and up to 80% of those hospitalised in critical care units [1]. Delirium is associated with negative clinical outcomes which include longer hospitalisations, increased direct and indirect costs, functional impairment, greater risk of mortality, increased risk of institutionalisation, and deterioration of long-term cognitive status, among others [1–4].

Subsyndromal delirium is a milder state characterised by the presence of certain delirium symptoms but without meeting full diagnostic criteria thresholds, is also associated with poor posthospitalisation outcomes similar to those associated with delirium [5]. The reported frequency of subsyndromal delirium varies (7–50%) according to the definition applied and the clinical population studied. Its incidence in the general medicine ward is 17% [6].

Delirium in hospitalised older adults is preventable. In multiple randomised clinical trials, non‐pharmacological interventions to prevent delirium (nPPD), shown to reduce its incidence by 30–50% [7]. These interventions include promoting physical activity, facilitating the use of glasses and hearing aids, and providing frequent temporospatial orientation and cognitive stimulation, among others [8, 9]. Despite the high evidence supporting them, nPPD are poorly implemented in hospitals in Chile and around the world for several reasons, including the heavy workload of clinical staff, lack of trained personnel, and in general, the absence of systematic implementation processes [10, 11]. The professional who can more systematically promote cognitive stimulation at the hospital is the occupational therapist. It has been seen that delirium has been prevented in non-ventilated older adults who receive therapy twice a day [9]. However, considering the large number of hospitalised older adults and the number of interventions required for each patient, this alternative is sometimes not very accessible.

Before this clinical trial, local pilot studies have shown some progress in this area. It has been reported that the incorporation of software technology through a "PREVEDEL" tablet is feasible and can be implemented on a hospital level, with possible effects on decreasing delirium [12] and the implementation of nPPD following a theoretical model improves the adherence of health personnel [13].

We propose a multicentre randomised clinical trial to evaluate whether cognitive stimulation guided by PREVEDEL software prevents delirium in hospitalised older adults and whether it is a technology that can be used in others hospital centres. The principal aim is to compare the incidence of delirium and subsyndromal delirium in hospitalised older adults using cognitive stimulation guided by PREVEDEL software versus nPPD.

## Methods and design

### Study design

This will be a multi-centre, parallel-group, randomised controlled trial (RCT). CONSORT methodological recommendations will be used for reporting non-pharmacological trials, in addition to Templates for Intervention Description and Replication and Standard Protocol Items: Recommendations for Interventional Trials. This project was registered at ClinicalTrials.gov, the identifier is NCT05108207.

### Participants

The research is developed in four hospital centres of similar complexity located in Santiago, Chile. These are: Hospital Clínico de la Universidad de Chile (HCUCH), Hospital San Juan de Dios, Clínica Las Condes, and Hospital Santiago Oriente Dr. Luis Tisné. Two are public institutions and two are private institutions. Patients hospitalised in general wards (Geriatric and Internal Medicine Units) and Intermediate Care Units. The units serve older adults admitted for acute pathologies, in the intermediate care unit patients can receive non-invasive ventilation, high-flow nasal cannula, and low doses of vasopressors.

We will include patients 65 years or older, hospitalised less than 48 h prior in a general ward or the intermediate care unit of four participating centres. Patients with full or syndromal delirium (Confusion Assessment Method-[CAM] + or presence of one core element) history of dementia (Alzheimer’s Disease-AD8 greater than 2 points), and non-Spanish speaking patients will be excluded.

### Interventions

Patients will be randomised to one of the following groups: (i) the control group: will receive the standard delirium prevention measures outlined above, plus the use of a mobile device without delirium prevention software for five days (control group: nPPD package, without PREVEDEL) or (ii) the experimental group: will receive the standard delirium prevention measures, plus the use of a mobile device with PREVEDEL software for five days (experimental Group: nPPD package, with PREVEDEL).

A) Preparation of interventions to implement a programme of nPPD in the centres.

Three months before the start of the clinical trial, an intervention will be conducted in the internal medicine, geriatrics, and intermediate care unit services of the four participating centres to establish a homogeneous plan of nPPD and assess adherence. The implementation methodology in the first stage will follow the Consolidated Framework for Advancing Implementation Science model, used in a local pilot study [13]. The following actions are considered:

- To assess healthcare personnel's knowledge and beliefs about delirium. The same questionnaire is used as in the previous pilot study of implementation in HCUCH [13].
- Define local leaders and roles. Leaders are designated according to interest and motivation to lead the implementation of the protocol in their unit with the aim of incorporating the proposed activities into the daily care routine. These leaders will have meetings for feedback and support.
- Delivery of study material.
- Approval of online course on delirium and nPPD.
- Assessment of adherence to four non-pharmacological prevention domains (12 indicators): Management of environment (visible clock, visible calendar and decrease physical restrictions) early mobilisation (indication of relative rest, encourage the patient to sit and stand), correction of sensory deficits (availability of glasses, hearing aids, and dentures when necessary), management of medications (decrease the use of benzodiazepines, anticholinergics, and metoclopramide when possible) [13]. The control and experimental groups will have four non-pharmacological prevention domains.
- Provide an informative diptych on delirium for the patients and families.

B) Use a mobile device. We will use an Alcatel OneTouch Pixi-3 10 tablet which will be delivered daily to each patient between 9:00 am and 8:00 pm, with nighttime pickup (8:00 pm to 09:00 am), so the patient can rest and to charge the device. On the first day, a previously trained health professional will deliver the tablet and train the patients in its use, taking 10–15 min. The day after the tablet is delivered, the same person will go to the patient's room to check if he or she has any questions or problems with its use. Both groups are advised to use the tablet three times a day, for at least ten minutes each time.

To evaluate patient adherence and how the tablet is used, both groups will have an application installed in their devices that will allow them to keep track of the duration of use and applications used.

C) The control group will receive the standard delirium prevention measures outlined above, plus the *use of a mobile device without delirium prevention software* for five days (Control Group: nPPD package, without PREVEDEL).
D) The experimental group will receive the standard delirium prevention measures, plus the use of a mobile device with PREVEDEL software for five days (experimental Group: nPPD package, with PREVEDEL).
E) The PREVEDEL software was designed and evaluated in a previous study in Chile. It proved to be highly accessible and feasible to improve access to nPPD in hospitalised older adults and showed its possible effect in decreasing the incidence of delirium [12].

To define the software content, a search was carried out in the literature of systematic reviews on non-pharmacological prevention of delirium. The data were reviewed, and the multidisciplinary team chose specific interventions based on their evidence and the feasibility of their incorporation into the software. A literature search was conducted to define the design characteristics of the software and to optimise accessibility for older adults. The data were reviewed, and the multidisciplinary team selected specific design features based on their clinical experience, the technical feasibility to be incorporated into the software, and the opinion of the older adults who participated in the software development.

The software developed was organised into several modules for easy access, which include:

A) Desktop module: this is the first screen the user faces when the software is turned on. It continuously delivers information for temporal and spatial reorientation and allows access to other modules.
B) The exercise module contains 12 physical activity videos guided by physical therapists, which can be easily performed by older adults. All exercises are focused on functional activities.
C) Games module: includes eight cognitive activities to stimulate attention, memory, and executive function. Colloquial names were selected to improve accessibility. Two different categories of difficulty were considered, according to the patient's level of education based on the above data [14].
D) The documentary module contains short videos intended to be a tool for cognitive stimulation, an entertainment activity during free time, and a tool to educate about delirium.
E) Alert module: a series of pre-set alerts are displayed during the use of the software. These include the promotion of sensory support measures (glasses, hearing aids), assessment of pain level, and sleep recommendations, with a customisable frequency.
F) Configuration module: allows software customisation. It is possible to configure patient data, including educational level, the use of sensory support, and restriction of patient movement if indicated. Access to this module can be password-protected. Healthcare personnel, especially nurses, can use this module to customise the software according to each patient's profile. For example, if the patient has an indication for absolute rest, it is possible to hide all physical activity videos, and if there is a ban on aerobic activity, only exercises with low metabolic demand are shown.
G) Registration system. The software keeps a complete record of its use, including the total duration of use and the duration for each module. It also records the number of correct or incorrect answers for cognitive games.

### Outcomes

A) Primary outcome:

To evaluate the incidence of delirium and incidence of subsyndromal delirium by applying the CAM [15] twice daily during five days of hospitalisation.

The incidence of basal delirium will be determined before the software is implemented in a group of patients enrolled prospectively with the same inclusion/exclusion criteria and delirium assessment protocol.

B) Secondary outcomes:
  Duration of delirium (days) and density of delirium (incidence x duration) will be assessed with the CAM.
  Level of independence in basic activities of daily living on day 5, will be assessed using the Functional Independence Measure (FIM), this instrument evaluates independence in 18 activities of daily living (13 motor and five cognitive items). The scale has seven levels (1–7, from lower to higher levels of independence). The motor FIM score ranges from 13–91, cognitive FIM from 5–35 points, and total FIM from 18–26 points; higher scores indicate greater independence [16].
  Cognitive level on Day 5 assessed using the Montreal Cognitive Assessment. The test evaluates attention and concentration, executive functions-memory, language, visuoconstructional skills, conceptual thinking-calculations, and orientation. It has Chilean validation in the population over 60 years of age [17].
  Hospital stay (days).
  90-day mortality.
  Duration of device use will be compared using the internal device registration.
  Presence of sleep disorders and headache will be evaluated using a self-reporting instrument in both groups. The doctor in charge of the patient will be informed for management.
  Adherence to the nPPD programme will be evaluated in the four participating centres before and after training in prevention measures. Assessment of adherence to non-pharmacological prevention domains (12 indicators) will be conducted [13].

In addition, socio-demographic data and clinical information will be gathered including The reason for admission, the Acute Physiology and Chronic Health disease Classification System II score, the Sequential Organ Failure Assessment), and the Charlson Comorbidity Index.

### Study sample

**Fig. 1:**
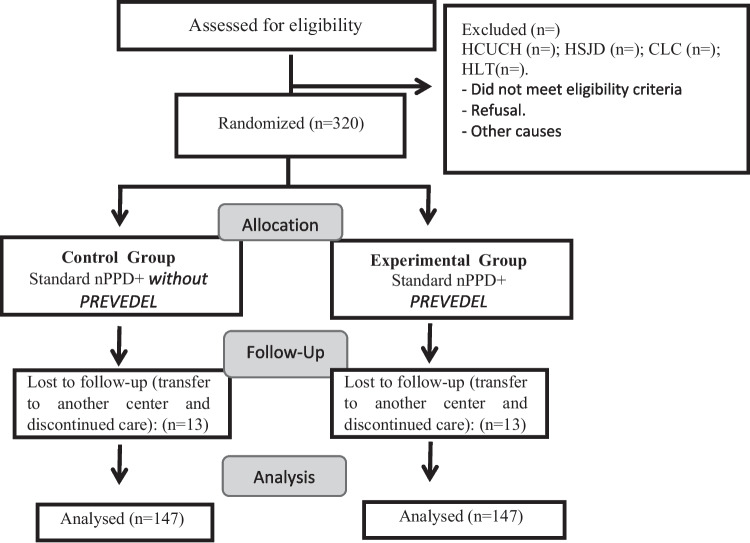
Patient flow chart

Local and literature data were evaluated to calculate the sample size [1, 13]. A delirium status prevalence of 20% is estimated in the control group and a decrease in prevalence to 8% in the intervention group is expected. In this way, with a power of 80%, and an alpha of 0.05 bilaterally, 147 patients will be enrolled in each group. If we add to this a 10% loss of follow-up, the overall sample size will be 320 patients (See flow chart in Fig. 1).

### Interim analysis

The Trial Steering Committee (TSC) was formed with three international experts in the area and will ensure the following aspects:

- Supervise and guarantee scientific quality.
- Advise on study protocol, recruitment progress, adherence, follow-up, and data quality.
- Review the recommendations of the data analysis and monitoring committee.
- Review the publications in the primary trials.

For the fulfilment of the objectives, the TSC will meet before, in the middle, and at the end of patient recruitment.

### Randomisation

Randomisation will be performed using computer-generated random permuted blocks using a centralised, secure, Web-based randomisation service. A computer will assign blocks of ten for each centre so that in each of the four participating centres, there will be a similar number of patients in each group.

### Management, implementation and masking

All the centres have geriatric units and a rehabilitation team that includes a physiatrist, kinesiologist, occupational therapist, and speech therapist. All centres have delirium prevention protocols, one of them with formal research in the implementation of nPPD and use of the software (pilot studies) [12, 13]. Each centre has a specific coordinator and implementing staff. All care providers will take an online course in non-pharmacological management of delirium that will be led by a multidisciplinary research team (physicians, nurses, occupational therapists, and engineers). This will be conducted before recruitment to achieve a homogeneous standard of treatment. In addition, they will participate in telematic meetings to disseminate information, get feedback to increase adherence.

When the patient signs the informed consent, the recruiter will give notice to an independent statistician who will make the random assignment and inform the tablet coordinator via text message, the coordinate will be in charge of installing or not the PREVEDEL software in the tablet according to the assigned group. This person will not have any contact with the patients. A healthcare professional (doctor or occupational therapist) will deliver the tablet and train the patient on how to use it.

The enrollers, evaluators, and healthcare personnel (nurses, physical therapists, physicians, occupational therapists) will be blind to the group assignments. Both groups will receive a tablet of the same dimensions and with the same basic applications. However, blinding the patient is not possible, as when exploring the tablet, he or she will recognise whether or not it has the PREVEDEL program included. To reduce this bias, the staff who delivers the tablet to the patients encourages both groups to use its different basic applications, that they access the internet, and that any questions they may have about its use should be addressed to the tablet coordinator so that the evaluators or healthcare personnel remain blinded.

The time between the assignment and delivery of the tablet will between 4–12 h in all participating centres. This variable will be measured for later analysis.

### Analysis and statistical methods

The results will be analysed with the intention to treat. For the descriptive analysis of the variables, we will use the mean (standard deviation), median (25–75 percentile), or proportions as appropriate to the type and distribution of the data.

Analytical Statistics: For the primary outcome, a comparison of proportions between patients who develop delirium and subsyndromal delirium in both groups will be made, using Fisher's exact test, and as a measure of efficacy, an odds ratio with 95% confidence interval will be estimated. If there is any baseline variable that is not matched by the randomisation, efficacy analyses will be conducted using logistic regression with the main variable being the treatment, and the adjustment variables will be those that the randomisation would not have been able to match. Additionally, to compare the effect of the intervention over time, a time-to-event analysis will be performed using the Kaplan-Maier procedure and then the Cox regression model to adjust for baseline variables and the presence of the intervention. For the secondary outcomes, the Fisher's exact test or t-test will be used, as appropriate for the type and distribution of the data. Linear regression or multivariate logistics will also be performed, as appropriate, to evaluate the impact of the intervention, adjusting for the input covariates. A bilateral *p* < 0.05 significance level will be used for all analyses.

## Discussion

The implementation of non-pharmacological prevention strategies for delirium is still rare in hospitals. This clinical trial seeks to enhance the implementation of these measures through the use of cognitive software (PREVEDEL) that allows older adults admitted to intermediate critical units and general rooms, to keep their cognitive functions active, promote early mobility, and the use of sensory devices to reduce the incidence of delirium and subsyndromal delirium. The use of PREVEDEL can increase the access to delirium prevention in hospitals, since there may be several patients using it simultaneously: relevant aspect in units with highly demanded professionals and with difficulties in the implementation of prevention measures [14, 18].

PREVEDEL is a software that has already tested its accessibility, usability, and acceptability in older adults, we will validate its use in this clinical trial [12].

Finally, if the results of this clinical trial are positive, it will be of great use to hospitalised patients and health professionals.

## Data Availability

Data sharing is not applicable to this article as no datasets were generated or analysed at this stage of the study. Once the study protocol is published, it will be shared and disseminated through clinical dissemination sessions (health personnel, undergraduate and postgraduate students) and on the website of the participating hospitals.

## Abbreviations

nPPD: Non‐pharmacological interventions to prevent delirium
RCT: Randomized controlled trial
HCUCH: Hospital Clínico de la Universidad de Chile
HSJD: Hospital San Juan de Dios
CLC: Clínica Las Condes
HLT: Hospital Santiago Oriente Dr. Luis Tisné
CAM: Confusion Assessment Method
AD8: Alzheimer Disease
FIM: Functional Independence Measure
TSC: Trial Steering Committee
FONDEF: Fondo de Fomento al Desarrollo Científico y Tecnológico
